# Factors Affecting Frequency Discrimination of Vibrotactile Stimuli: Implications for Cortical Encoding

**DOI:** 10.1371/journal.pone.0000100

**Published:** 2006-12-20

**Authors:** Justin A. Harris, Ehsan Arabzadeh, Adrienne L. Fairhall, Claire Benito, Mathew E. Diamond

**Affiliations:** 1 School of Psychology, University of Sydney, Australia; 2 Department of Physiology and Biophysics, University of Washington, Seattle, Washington, United States of America; 3 Cognitive Neuroscience Sector, International School for Advanced Studies, Trieste, Italy; Centre de Regulacio Genomica - Barcelona Biomedical Research Park, Spain

## Abstract

**Background:**

Measuring perceptual judgments about stimuli while manipulating their physical characteristics can uncover the neural algorithms underlying sensory processing. We carried out psychophysical experiments to examine how humans discriminate vibrotactile stimuli.

**Methodology/Principal Findings:**

Subjects compared the frequencies of two sinusoidal vibrations applied sequentially to one fingertip. Performance was reduced when (1) the root mean square velocity (or energy) of the vibrations was equated by adjusting their amplitudes, and (2) the vibrations were noisy (their temporal structure was irregular). These effects were super-additive when subjects compared noisy vibrations that had equal velocity, indicating that frequency judgments became more dependent on the vibrations' temporal structure when differential information about velocity was eliminated. To investigate which areas of the somatosensory system use information about velocity and temporal structure, we required subjects to compare vibrations applied sequentially to opposite hands. This paradigm exploits the fact that tactile input to neurons at early levels (e.g., the primary somatosensory cortex, SI) is largely confined to the contralateral side of the body, so these neurons are less able to contribute to vibration comparisons between hands. The subjects' performance was still sensitive to differences in vibration velocity, but became less sensitive to noise.

**Conclusions/Significance:**

We conclude that vibration frequency is represented in different ways by different mechanisms distributed across multiple cortical regions. Which mechanisms support the “readout” of frequency varies according to the information present in the vibration. Overall, the present findings are consistent with a model in which information about vibration velocity is coded in regions beyond SI. While adaptive processes within SI also contribute to the representation of frequency, this adaptation is influenced by the temporal regularity of the vibration.

## Introduction

Many investigations of sensory processing are based on the principle that the capacity to discriminate between two sensory stimuli must be based upon the difference between their neural representations. Thus, identifying how manipulations of a stimulus alter its percept can help elucidate the neural representation. The present work addresses the nature of neural coding in the somatosensory system: we have conducted psychophysical experiments to identify which features of a vibrotactile stimulus are extracted by the somatosensory system to determine its frequency, and which regions in the somatosensory cortical network are involved in this process.

Early investigations focused on the role of neurons in subcortical stations and primary somatosensory cortex (SI) in coding low frequency “flutter” vibrations (below 50 Hz) [1]–[3], while more recent work has emphasized the role of cortical areas “downstream” from SI, such as the second somatosensory cortex (SII) and regions of frontal cortex [4], [5]. Which of these different areas, and which features of the neural activity within these areas, are essential components in forming the percept of a vibration? A series of psychophysical experiments with humans provided evidence that neural processes in SI contribute to frequency discriminations. In a task designed to resemble that performed by monkeys in the aforementioned neurophysiological studies, subjects compared two sequential vibrations and reported which had the higher frequency. They became less accurate when the somatotopic distance between the two vibrations increased (ie, when the two vibrations were presented on different fingertips) [6]. We interpreted this drop in accuracy as evidence that SI neurons normally contribute to frequency discrimination when subjects compare two vibrations delivered to the same site. Because most SI neurons have contralateral receptive fields centered on a single finger[[7]–[9], but see [10], [11], [12] for recent evidence that SI receives ipsilateral inhibitory input in addition to contralateral excitatory input], those neurons would not receive input from somatotopically distant stimulus sites, and therefore would not be part of the substrate of frequency comparisons between fingers. Thus, when SI neurons were excluded from the discrimination process in this way, performance fell. This interpretation was confirmed in a subsequent experiment that investigated the effects of Transcranial Magnetic Stimulation (TMS) delivered to SI [13]. In that experiment, accuracy at comparing vibrations delivered to the same fingertip was reduced if neuronal activity in the contralateral SI was briefly interrupted by a TMS pulse delivered during the retention interval between the vibrations. It is worth noting that, although an increase in somatotopic distance and TMS both reduced discrimination sensitivity, they did not abolish it. This means that subjects also use information about vibration frequency coded in areas beyond SI that have bilateral receptive fields. The most obvious candidate area is SII. This is entirely consistent with the neurophysiological evidence that vibration frequency is coded by neurons in multiple cortical areas. Moreover, because different cortical regions encode vibrotactile stimuli in different ways [4], [5], the discrimination may be based on multiple features of the stimulus, each feature encoded most explicitly by a different cortical region.

If neurons in SI do contribute directly to the comparison of vibrations in a frequency discrimination task, what coding mechanisms might they use? The temporal structure of a vibration is explicitly represented in the precise phase-locked activity of SI neurons [1]–[3], and it has been suggested that the periodicity of this activity explicitly codes the vibration frequency [1]. However, this has been challenged by evidence from psychophysical and electrophysiological experiments with monkeys [4], [5], [14]. For example, Romo and colleagues reasoned that the addition of temporal noise to a vibration would create corresponding noise in the responses of SI neurons, and should therefore impair the frequency discrimination if the temporal structure of SI activity were used to code for frequency. After finding that the addition of such noise to the vibration did not reduce the monkeys' performance, they concluded that firing rate or spike count, but not spike timing, was the relevant code [14], [15]. We re-examine this issue in the present paper: We asked our human participants to compare vibrations whose temporal structure was altered by the addition of Gaussian noise (jitter) to the duration of each cycle of the sine wave. Moreover, if there is a temporal code for vibration frequency, and if this is represented in SI, then the effect of noise should be greater when subjects compare vibrations on the same finger than when they compare vibrations on different hands, because SI will contribute more to the former than to the latter comparison. Here, we test this prediction as well.

The present experiments also examined a second feature of vibrations that may contribute to perception of their frequency. Based on evidence that monkeys and humans perceive the “intensity” of a vibration to increase with its frequency [eg], [16, 17], the aforementioned studies with monkeys [1]–[5] eliminated subjective intensity as a cue to frequency by adjusting the amplitude of each vibration. In contrast, our previous experiments used vibrations with fixed amplitudes. This may be an important procedural difference if subjects do use subjective intensity as a cue when comparing vibration frequencies. Therefore, the present series of experiments examined this issue by investigating whether the perception of vibration frequency incorporates information that is sensitive to amplitude as well as frequency. However, rather than focusing on a subjectively defined entity – intensity – we have sought to quantify how amplitude and frequency are combined, so as to identify the relevant physical property of the vibration. The results indicate that the physical quantity corresponding to the product of amplitude and frequency is an important component of what people perceive as frequency.

## Results

### Experiment 1. The relationship between frequency and amplitude in frequency discrimination

If judgments of frequency depend purely on the temporal properties of a vibration, then performance will not be affected by alterations in vibration amplitude. Yet, it has often been assumed that humans and monkeys use the subjective intensity of a vibration, which is sensitive to amplitude as well as frequency, as a cue for frequency [16], [17]. Moreover, physiological experiments in rats [18], [19] show that cortical spike count – the neuronal correlate of perceived frequency according to Luna et al. [15] – is proportional to the product of frequency and amplitude. To investigate the relationship between amplitude and frequency in the perception of vibration frequency, Experiment 1 measured frequency discrimination thresholds while systematically varying the difference in the amplitudes (*Δ*A) of the two vibrations.

**Figure 1:**
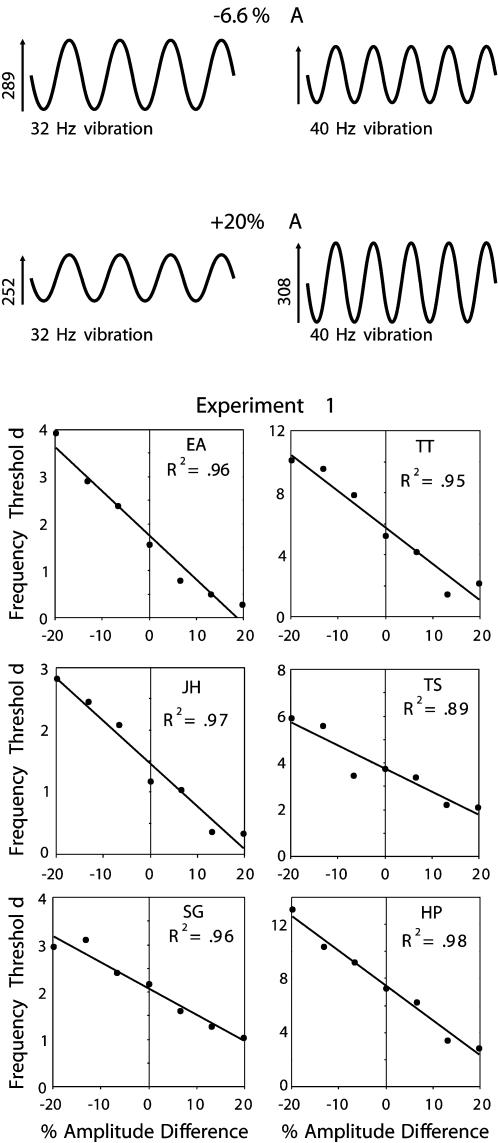
**Top**: Illustrations of the sinusoidal vibrations used in Experiment 1. Subjects compared the frequency of two vibrations that differed in amplitude (ΔA); the difference between the higher and lower frequency vibrations was either −20%, −13.3%, −6.6%, 0%, +6.6%, +13.3%, or +20%. The examples illustrated are for a 32 Hz and a 40 Hz vibration, with ΔA = −6.6% and +20%. **Below**: The six graphs plot the frequency discrimination thresholds (in Hz) for 6 different subjects as a function of ΔA. Each black point is the average threshold measured by two independently run adaptive staircases. Each graph includes the line-of-best-fit and the R^2^ for that regression line.

For each of the 6 subjects, the frequency threshold decreased as *Δ*A increased (see Figure 1), confirming that changes in amplitude can affect the perception of vibration frequency. In short, increasing the amplitude of a vibration increases its perceived frequency. Most pertinent to our hypothesis is the finding that the relationship between frequency discrimination threshold and *Δ*A was well approximated by a straight line. In 5 of the 6 subjects, the best-fitting line accounted for 95% or more of the variance of the data (R^2^ values ≥0.95); in the sixth subject it accounted for 89% of the variance. In other words, any increase or decrease in the amplitude of one vibration (within the tested range of ±20%) produced a proportional increase or decrease in its perceived frequency. Despite considerable individual variability in discrimination performance – baseline thresholds for vibrations of equal amplitude (*Δ*A = 0) varied between 1.2 Hz (subject JH) and 7.3 Hz (subject HP) – the effect of amplitude on perceived frequency was consistent across subjects. Therefore, when judging the frequency of a vibration, subjects are sensitive to the product of its amplitude and its frequency (*A*×*f*). This quantity corresponds to the root mean square (rms) velocity of the vibration, or, if squared, its energy. Experiment 1 thus quantifies earlier arguments that the perception of vibration frequency may be affected by the subjective “intensity” of the vibration [17]. Our findings also indicate that a vibration coding scheme identified in the whisker sensory system of rats [18], [19] may be general across species.

### Experiment 2: The contribution of rms velocity to frequency discrimination

Experiment 1 showed that, when judging the frequency of a vibration, subjects used the product of its frequency and amplitude (ie, the rms velocity of the vibration). Experiment 2 again measured the role of amplitude in frequency discrimination, this time in relation to the temporal retention interval separating the to-be-compared vibrations. This manipulation was included based on previous evidence that the involvement of SI neurons decreases progressively as the retention interval extends from 300 to 1200 msec [6], [13]. If velocity dependence showed a similar time course, we could posit SI as a crucial site contributing to the conversion of vibration velocity into the frequency percept. If velocity dependence showed a different time course, we would conjecture that regions beyond SI are as crucial to the representation of vibration velocity. Therefore, we measured discrimination thresholds for vibrations that either (1) had equal amplitude, such that rms velocity differed in proportion to the difference in frequency, or (2) had matched rms velocity (the vibrations differed in amplitude by the same proportion as they differed in frequency, but the higher frequency vibration had the smaller amplitude).

**Figure 2:**
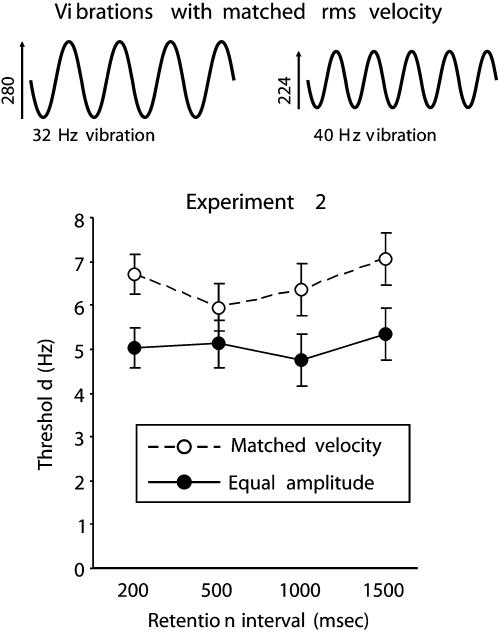
**Top**: Illustration of two sinusoidal vibrations with different frequency but equal rms velocity (proportional to *A*×*f*). **Below**: Results of Experiment 2 (vertical bars represent within-subject SEM). Subjects' thresholds for frequency discrimination were higher when comparing two vibrations with matched velocity than for vibrations with matched amplitude (and thus different velocity). This greater difficulty in discriminating matched-velocity vibrations did not vary systematically as the retention interval increased.

The results of Experiment 2, shown in Figure 2, confirm the basic finding of Experiment 1. When comparing vibrations with matched velocity, the subjects' thresholds were on average 30% higher than when they compared vibrations with the same amplitude. This overall difference was significant (*t*_19_ = 4.04, *p*<.001), but it did not vary systematically with increases in the retention interval ranging from 200 to 1500 msec (*F*s ≤ 1.04 for all interactions between vibration type and retention interval). Subjects appeared to use the vibration velocity across intervals that were longer than those spanned by an SI frequency code [6], [13], suggesting that information about rms velocity may be distributed in a network extending beyond SI. This possibility was investigated further in Experiment 3.

### Experiment 3: The contribution of rms velocity to unilateral versus bilateral frequency comparisons

We have previously shown that subjects are more accurate at comparing vibrations presented on the same finger than vibrations presented on opposite fingers [6], [13], an effect we attributed to the contribution provided by neurons with unilateral receptive fields (such as generally characterizes neurons in SI) since this contribution would be available for same-finger comparisons but unavailable for opposite-finger comparisons. Nonetheless, the fact that subjects can compare vibrations on opposite hands indicates that the frequency comparison also occurs in areas beyond SI, consistent with the evidence reported from electrophysiological studies with monkeys [4], [5]. Therefore, if information about velocity is held by neurons in cortical regions beyond SI, as suggested by Experiment 2, then subjects should depend on velocity when discriminating between vibrations presented to opposite fingers. That is, the increase in frequency threshold that occurs when subjects compare vibrations with matched velocity should be equivalent for same-finger and opposite-finger comparisons. Experiment 3 tested this hypothesis.

**Figure 3:**
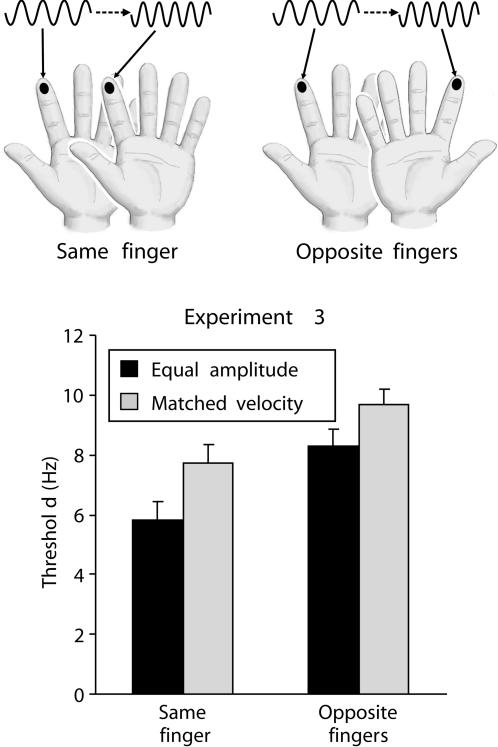
**Top**: Illustration of the design of Experiment 3, in which two vibrations were either presented on the same index finger (both left or both right) or were presented on opposite index fingers. **Below**: Results for same-finger and opposite-finger comparisons of vibrations that either had matched amplitude (and thus differing velocity) or had matched velocities (vertical bars represent within-subject SEM). The subjects' ability to discriminate the frequency of the two vibrations decreased (their discrimination threshold increased) if the vibrations had matched velocity, or if the vibrations were presented to opposite fingers, and these two effects were additive in that the subjects performed worst when comparing matched-velocity vibrations presented on opposite fingers.

The subjects' discrimination thresholds were higher when comparing vibrations on opposite fingers than when comparing vibrations on the same finger (Figure 3; *F*_1,19_ = 61.08, *p*<.001), confirming previous studies [6], [13]. Thresholds were also higher when comparing vibrations with matched rms velocity than vibrations with equal amplitude (*F*_1,19_ = 11.46, *p* = .003), confirming Experiment 2. However, the most important finding is that the effect of matching rms velocity was equivalent for both same-finger and opposite-finger comparisons – there was no interaction between topography (same versus opposite fingers) and velocity (*F*<1). Paired *t*-tests confirmed that there was a significant difference between equal-amplitude and matched-velocity vibrations for both same-finger and opposite-finger comparisons (*t*_19_ = 3.03 and 2.47, *p* = .007 and .023). We conclude that subjects use rms velocity when judging vibration frequency even when neurons with unilateral receptive fields (presumably in SI) are excluded from the direct comparison process.

### Experiment 4: The combined effects of temporal noise and rms velocity on frequency comparisons

**Figure 4:**
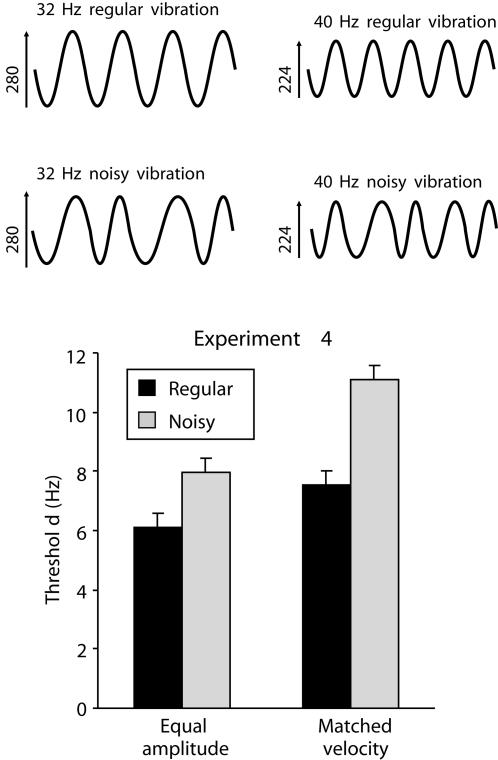
**Top**: Examples of periodic and noisy sine wave vibrations used in Experiment 4. Noisy vibrations were created by adding a positive or negative random interval (from a Gaussian distribution with mean = 0) to the length of each cycle of the regular sine wave (from 0° to 360°). For example, for a 40 Hz noisy vibration, the length of each cycle varied randomly around a mean of 25 msec. Note, the end of the last cycle was fixed, so that the vibration always had a total duration of 1000 msec. In the examples shown, two vibrations differing in frequency by 8 Hz also differed in amplitude such that they had matched velocity. **Bottom**: Results of Experiment 4 in which subjects compared two periodic or two noisy vibrations that either had the same amplitude or matched velocity (vertical bars represent within-subject SEM). Frequency discrimination was worse (thresholds increased) if the vibrations were noisy or had matched velocity, and these two effects combined super-additively, in that noise increased thresholds more when subjects were comparing vibrations with matched velocity than when comparing vibrations with equal amplitude.

It has been suggested that the phase-locked firing pattern of SI neurons constitutes a neural code for vibration frequency [1], which implies that the accuracy of frequency perception should be sensitive to noise in the temporal structure of the vibration. This idea was challenged by the finding that noise did not affect frequency discrimination in monkeys [14], leading to the opposite conclusion that monkeys do *not* use spike-timing information to perform frequency discrimination. However, our own recent observations indicate that noise can impair frequency perception in humans [20]. A potentially important difference between the studies was that Romo and colleagues tested monkeys with vibrations that were matched for subjective intensity (and thus presumably rms velocity) whereas we used vibrations that had equal amplitude (and thus differed in rms velocity). Experiment 4 investigated the potential role of temporal structure. Subjects compared the frequency of vibrations whose temporal structure was altered by the addition of Gaussian noise (jitter) to the duration of each sine-wave cycle (illustrated in the upper panel of Figure 4). The presence or absence of noise was combined in a factorial design with the presence or absence of differences in rms velocity (subjects compared vibrations that either had matched velocity or equal amplitude, as in Experiments 2 and 3). All vibrations were presented to the same finger.

Frequency discrimination was impaired by the addition of noise to the temporal structure of the vibrations (Figure 4, lower panel; *F*_1,15_ = 28.17, *p*<.001). Frequency discrimination was also impaired when the rms velocity of the two vibrations was matched (*F*_1,15_ = 21.26, *p*<.001). Of most interest, the effects of noise and matching velocity appeared to be super-additive, revealed by a significant interaction between the two factors (*F*_1,15_ = 6.21, *p* = .025). This super-additive interaction indicates that, when rms velocity was available as a correlate of vibration frequency, subjects were *less* sensitive to the impact of temporal noise; when velocity was removed as a cue for frequency, frequency discrimination became particularly vulnerable to the impact of noise.

### Experiments 5 and 6: The relationship between temporal noise and somatotopic distance

The experiments presented so far suggest that frequency discrimination relies on a network of cortical regions (SI and higher-order areas) and that the vibration features that contribute to the judgment of frequency include rms velocity and temporal structure. The two final experiments in this series employed a 2×2 factorial design to investigate the interaction between somatotopic distance and the presence of temporal noise. The two experiments differed in the availability of rms velocity as a correlate of frequency: in Experiment 5 the vibrations had fixed amplitude such that their rms velocity covaried with frequency; in Experiment 6 the amplitudes were manipulated so that the vibrations had matched rms velocity.

**Figure 5:**
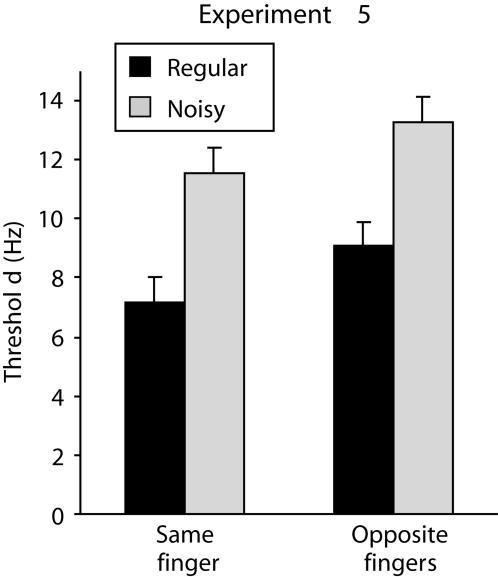
Results of Experiment 5 in which subjects compared two periodic or two noisy vibrations that were presented to either the same index finger or to opposite index fingers (vertical bars represent within-subject SEM). The vibrations all had fixed amplitude. Frequency discrimination was worse (thresholds increased) if the vibrations were noisy or were presented to opposite fingers, and these two effects combined additively in that the subjects' thresholds were close to that computed to arise from the effects of noise and matched velocity combined independently.

In Experiment 5 (see Figure 5) there was a significant difference between the thresholds obtained for same-finger and opposite-finger comparisons (*F*_1,9_ = 5.51, *p* = .044), and a significant difference between the thresholds obtained with regular versus noisy vibrations (*F*_1,9_ = 26.51, *p*<.001), confirming the findings of Experiments 3 and 4. There was no interaction between the effects of somatotopic distance and noise (*F*_1,9_<1, *p* = .91), indicating that same-finger and opposite-finger comparisons were equally sensitive to the effect of noise in the temporal structure of the vibrations.

**Figure 6:**
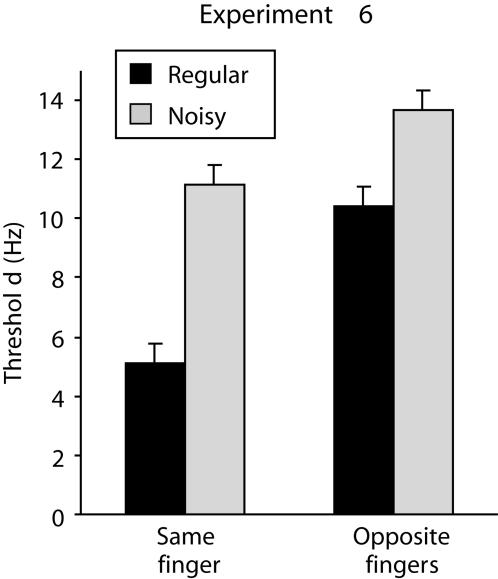
Results of Experiment 6 in which subjects compared two periodic or two noisy vibrations that were presented to either the same index finger or to opposite index fingers (vertical bars represent within-subject SEM). The vibrations all had matched rms velocity. Frequency discrimination was worse (thresholds increased) if the vibrations were noisy or were presented to opposite fingers, but these two effects combined sub-additively, in that the effect of noise was smaller when subjects were comparing vibrations on opposite fingers than when subjects compared vibrations on the same finger.

In Experiment 6 (see Figure 6), as in Experiment 5, there was a significant effect of somatotopic distance between the vibrations (*F*_1,9_ = 13.40, *p* = .005) and a significant effect of noise (*F*_1,9_ = 27.28, *p*<.001). However, unlike Experiment 5, there was a significant interaction between the effects of somatotopic distance and noise (*F*_1,9_ = 8.52, *p* = .017). As is evident in Figure 6, this interaction reflects the fact that noise had a greater effect on discrimination thresholds for same-finger comparisons than for opposite-finger comparisons. This implies that, once rms velocity differences between vibrations have been eliminated, the contribution made by SI neurons to the frequency discrimination is particularly sensitive to the effect of noise in the temporal structure of the vibration. The fact that this interaction was observed in Experiment 6, but not in Experiment 5, suggests that subjects are flexible in their use of different coding mechanisms: their reliance on temporal structure increases when velocity information is no longer available.

## Discussion

These experiments show that the ability of human subjects to compare the frequency of two vibrations is diminished by 3 manipulations: (i) increasing the somatotopic distance between the vibrations; (ii) adjusting the amplitudes of the vibrations to offset differences in their rms velocity (or energy); and (iii) adding noise to the temporal structure of the vibrations. We will discuss each of these effects in turn.

The effect of somatotopic distance was reported in our previous work [6], [13], and has led us to conclude that neurons with small unilateral receptive fields (such as those in SI) can make a direct contribution to the process by which the frequency of the first vibration is remembered and compared with that of the second vibration. The performance difference in relation to somatotopic distance was present for retention intervals of up to 800 msec, indicating that the direct involvement of SI in the retention of frequency information is temporally limited. The finding that performance decreases as somatotopic distance increases is open to other interpretations: for example, one might posit difficulty in shifting attention between fingers. This possibility, however, is discounted by the fact that the very time window across which the somatotopic effect is observed (200 to 800 msec after the first vibration) corresponds to the time course of “inhibition of return” effects – people are slower at detecting a tactile signal if it is preceded by an ipsilateral cue rather than a contralateral one [eg], [21, 22]. Therefore the decrease in frequency discrimination with increasing somatotopic distance arises in spite of attention effects rather than because of them. Another possible explanation for the somatotopic effect is that the SI response to the second vibration is inhibited by ipsilateral input resulting from the first vibration [10]–[12]. However, there is currently no evidence that the inhibitory input to the ipsilateral SI outlasts the presentation of the stimulus itself, as would be required to explain the present findings. Moreover, in addition to the evidence from somatotopy, support for the conclusion that SI is directly involved in vibration comparisons comes from the finding that a TMS pulse to SI interferes with frequency discrimination in humans [13]. Moreover, the evidence obtained with TMS matches the evidence from somatotopy in identifying the time course of SI involvement.

It is clear that frequency discrimination in humans does not rely solely on SI because, when neurons in SI were excluded from the comparison task through bilateral stimulus presentation, subjects were still able to compare frequency, albeit with lower sensitivity. Thus areas downstream from SI, with bilateral receptive fields, must also contribute to frequency discrimination in humans, as they do in monkeys [23], [24], and as they do for other tactile discrimination tasks in humans [25]. The present experiments confirm this conclusion and additionally reveal that these areas use, in part, the rms velocity of the vibration to code for its frequency. In Experiment 1, the threshold to perceive a difference in frequency between two vibrations was *linearly* affected by the difference in their amplitudes. Therefore, the perceived frequency depended on the product of amplitude and frequency, (*A*×*f*)^n^, corresponding to the rms velocity of the vibration if n = 1, or to its energy if n = 2. Accordingly, when the difference in velocity of the two vibrations was eliminated, the subjects were less sensitive in discriminating their frequencies. Moreover, this effect was observed for both same-finger and opposite-finger comparisons, indicating that rms velocity is represented by neurons with bilateral receptive fields, pointing to the involvement of regions beyond SI.

The evidence that human subjects used rms velocity as an index of vibration frequency corroborates earlier arguments that frequency perception involves subjective “intensity”, a quality that is sensitive to amplitude [eg, 17]. It is also consistent with recent electrophysiological studies of SI neurons in rats [18], [19]: the firing rate of SI neurons during delivery of sinusoidal vibrations to the whiskers did not explicitly encode the frequency or amplitude of the vibration, but did encode their product, (*A*×*f*)^n^. The fact that humans also detect rms velocity implies that, for this kind of stimulus, tactile coding strategies are general across species.

The third manipulation shown to affect frequency discrimination was the addition of noise to the temporal structure of the vibrations. Noise interacted with both of the other factors studied here, somatotopy and rms velocity. First, noise was more disruptive when added to vibrations that had matched velocity than to vibrations with equal amplitude (and thus different velocity). We interpret this to mean that, when differences in rms velocity were available to aid in frequency discrimination, subjects were less sensitive to the effects of temporal noise. This suggests that the coding of rms velocity offers a channel of frequency information that is relatively robust against variability in the temporal structure of the vibration, as might be expected if some temporal averaging is taking place in the representation of velocity (indeed this is implied by “rms”). Second, noise was less disruptive for frequency judgments of vibrations presented on different hands than for vibrations presented on the same finger, as long as the vibrations had matched rms velocity. Therefore, a second coding channel for vibration frequency (distinct from velocity) is particularly sensitive to noise in the temporal structure of the vibrations and appears to be identified with neurons that have unilateral receptive fields, such as characterizes neurons in SI. This is consistent with the neurophysiological evidence from monkeys that neuronal activity in SI is entrained to the pattern of a vibrotactile stimulus – information about vibration frequency could thus be “read off” from this activity, either as the inter-spike intervals [1] or the number of spikes [15].

If vibration frequency is, in part, coded by the precise temporal structure of neural activity in SI, an obvious mechanism by which SI neurons could contribute to frequency discriminations would be for those neurons to maintain their phase-locked activity during the retention interval, so that the inter-spike intervals induced by the first vibration could be compared against those induced by the second vibration. Noise in the temporal structure of the vibrations would introduce noise in the inter-spike intervals, thus impairing frequency discrimination. However, numerous neurophysiological investigations by Romo and colleagues uncovered no evidence for sustained activity in SI. Moreover, the response of SI neurons to the second vibration is unaffected by the frequency of the first vibration [4], [5], which is inconsistent with the suggestion that these neurons code the difference in frequency. On these grounds, Romo and colleagues have argued that vibration frequency is represented in SI by firing rate or spike count rather than phase-locked inter-spike intervals [5], [15], [23], [26]. Our data are open to this alternative hypothesis if, as discussed below, noise in the temporal structure of the vibration also affects firing rate.

Romo and Salinas [24] suggested that adaptation of neuronal activity in SI may account for the evidence of SI involvement in frequency discrimination in humans. Adaptation in SI neurons during the first vibration could reduce variability of their response to the second vibration if that vibration occurs soon after the first vibration [see reference 27 for evidence of carry–over adaptation in SI between sequential vibrations]. The resultant increase in the fidelity of the SI response would improve frequency discrimination. Consistent with our present and past findings [6], [13], this adaptation effect would be somatotopically specific (showing no transfer between hands), and would likely be disrupted by TMS. Consistent with physiological findings [4], [5], the adaptation effect would not be expected to produce spiking activity among the SI neurons during the retention interval. The current findings indicate that the proposed adaptation effect in SI is sensitive to the temporal structure of the stimulus, in that SI neurons must adapt more effectively to periodic vibrations than noisy vibrations. Evidence supporting this conclusion has been obtained recently from electrophysiological recordings in rat somatosensory cortex during trains of whisker stimulation [28]. The strength of adaptation was reduced (allowing steady-state firing rate to remain higher) if the sequence of deflections had a noisy temporal structure. This implies that the mechanism of adaptation is more strongly engaged by periodicity in the temporal structure of the sensory signal [see 29], [30].

Differential engagement of adaptive mechanisms by periodic versus noisy stimulus trains could help explain the range of findings reported here. We postulate that the adaptation of SI neurons during periodic stimulus trains steepens the input-output function relating vibration frequency to firing rate around the frequency of the adapting stimulus, and thereby improves frequency discrimination. A reduction in this adaptation process is responsible for the lower discrimination accuracy with noisy vibrations. If two periodic vibrations are presented to the same finger, such that the second vibration engages the same neurons that have adapted to the first vibration, frequency discrimination thresholds should be particularly low. A recent functional magnetic resonance imaging study in humans confirmed that neurons in SI remain adapted to a periodic vibrotactile stimulus across a temporal interval of 600 msec [27]. If the second of two periodic vibrations is presented on the opposite hand, it will engage a new and unadapted population of SI neurons in the opposite hemisphere, causing discrimination thresholds to be higher. Nonetheless, the adaptation of SI neurons in different hemispheres will still improve discrimination by providing a more reliable signal to downstream areas with bilateral receptive fields (eg, SII). Therefore, by impairing adaptation, noise will still reduce discrimination of vibrations on different hands, but the net impact of noise should be smaller than for same-finger comparisons where there is the added benefit of carry-over adaptation between vibrations.

The proposed adaptation mechanism may also account for the conflicting evidence concerning the effects of noise. While the present experiments have shown that noise degrades frequency discrimination in human subjects, previous studies with monkeys, using a protocol very similar to that employed here, found that the addition of noise to the vibrations had no effect on the monkeys' performance [14], [15]. As we have noted previously [13], a significant difference between the two sets of studies is that the monkeys tested by Romo and colleagues were given several months of training on the task, whereas our human subjects were given no previous training at all. Extensive training with vibrotactile stimuli has been shown to induce lasting changes in the response of SI neurons [31], [32]; this training effect could reflect a relatively stable adaptation state among the neurons in SI. Once the neurons are stably adapted, they may no longer show transient adaptive changes in response to single periodic vibrations, thereby removing the basis on which noise impairs discrimination performance.

### Conclusion

The current experiments showed that information about vibration frequency was available in two distinct forms: as a representation of the vibration's rms velocity (or energy), which was degraded when velocity cues were removed; and as information that was sensitive to the vibration's temporal structure that could be degraded by noise. Once velocity cues were removed, the impact of noise was larger when subjects compared vibrations delivered to a single fingertip than when they compared vibrations delivered to fingers on different hands. By contrast, rms velocity contributed equally to the frequency discrimination when vibrations were on the same finger or opposite fingers. These observations identify the effects of noise with a representation of vibration frequency in SI, and are consistent with suggestions that the representation of frequency is improved by adaptation of neurons in SI, and that this adaptation process is driven by periodicity of the vibrotactile stimulus. Because evidence for a velocity code was obtained with both single-finger and opposite-finger comparisons, we conclude that this velocity is coded by neurons with bilateral receptive fields, such as in SII. Thus, vibration frequency is coded by multiple mechanisms distributed across multiple cortical regions, and the degree that each mechanism contributes to the perceived frequency depends on the information present in the vibrations and their relative locations on the body.

## Methods

### Materials

In Experiments 1, 2, and 4, the subject's right index finger pad rested on a 3-mm diameter steel rod. The rod was driven by a small vibration excitor and power amplifier (Type 4810 “minishaker”, and Type 2718 amplifier, Brüel & Kjaer, Denmark). This setup could only be used to present vibrations to a single finger. Therefore, for Experiments 3, 5 and 6 in which vibrations were presented on opposite fingers, stimulators were built using nickel bimorph wafers (38×19×0.5 mm, length×width×thickness; Morgan Matroc, Bedford, OH, USA). The wafers were individually mounted on plastic blocks, aligned side-by-side and spaced 25-mm apart (center-to-center), housed inside a custom-built case. Vibrations, presented to the pad of one or both index fingers, were transmitted by a 3-mm diameter plastic rod glued to the top face of a wafer. For both types of stimulator, the timing and waveform of the vibrations were controlled from a computer running Labview software (National Instruments, Texas).

### Procedure

The tactile stimuli were 1-sec long sinusoidal vibrations with base frequency of 32 Hz and base amplitude of 280µ. On each trial the subjects compared two consecutive vibrations, separated by a given retention interval, delivered to their right or left index finger. One of the vibrations was 32 Hz; the other varied from trial to trial (according to an adaptive staircase procedure, described below) but was always greater than 32 Hz. The order of the two vibrations was random from trial to trial, and the subjects had to report whether the second vibration frequency was higher or lower than the first. This design ensured that the subjects compared the two vibrations, rather than being able to make a categorical judgment about the frequency of the second vibration independently of the first [33]. The subjects were never given feedback on their response.

Each subject's frequency sensitivity was measured using an adaptive staircase procedure which automatically tailors the task difficulty to individual performance, making the test less vulnerable to ceiling or floor effects, and keeping the task difficulty constant across all experimental conditions. On each trial, the difference in frequency, *Δ* *f*, between the two vibrations was initially set at 8 Hz and then progressively decreased or increased across trials depending on whether the subject responded correctly or incorrectly on the previous trial. In Experiments 2 to 6, the value of *Δ* *f* on trial *n* was determined by the equation:
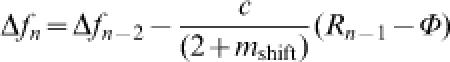
where *c* is a constant (set at 8), *m*_shift_ is the number of reversals so far (a reversal occurs whenever *Δ* *f* changes sign), *R*_n−1_ is the response in the previous trial (1 for a correct response and 0 for a false response), and Φ is the probability value to which the staircase should converge (set at 0.8125). The staircase was run for 12 reversals, and the threshold was calculated as the average *Δ* *f* across the last 6 reversals. In Experiment 1, *Δ* *f* was varied according to a Bayesian adaptive procedure that optimizes the information gain on each trial, and thus can be used to obtain efficient estimates of sensitivity threshold from a 30-trial staircase [34]. This Bayesian procedure was not used in the other experiments because, in our experience, the approach does not provide reliable estimates of threshold in psychophysically-inexperienced subjects (such as those tested in Experiments 2 to 6), probably because these subjects have high and variable lapse rates.

Recruitment of subjects and all experimental procedures were approved by the institutional ethics committee.

Experiment 1 investigated how information about the amplitude of a vibration is incorporated into the perception of its frequency. The 6 participants (two males, authors JH and EA, and four females who were naïve to the purposes of the experiment) ranged in age from 20 to 39 years, and one was left handed. Subjects compared the frequency of two vibrations that had either the same amplitude, or differed in amplitude by varying amounts. The difference in amplitude (*Δ*A) between the higher and the lower frequency vibrations was −20%, −13.3%, −6.6%, 0%, +6.6%, +13.3%, or +20% relative to their mean amplitude, where positive differences mean that the higher frequency vibration had higher amplitude. Test sessions consisted of multiple intermixed staircases with different *Δ*As. For each subject, the frequency discrimination threshold for each of the 7 *Δ*A conditions was the average of 2 separate staircases.

Experiment 2 investigated whether people use information about rms velocity (or any other measure proportional to A×*f*, such as energy) when judging vibration frequency. There were 20 subjects (14 females) aged between 18 and 37 years, and one was left handed. They compared the frequency of two vibrations that had either the same amplitude (so that rms velocity covaried with frequency) or the same rms velocity (the amplitude of the higher frequency vibration was reduced in proportion to the difference in frequency, see Figure 2). We also investigated whether the impact of this manipulation changed as the retention interval between the two vibrations increased. Subjects were tested with 4 different retention intervals: 200, 500, 1000, and 1500 msec.

Experiment 3 investigated whether the somatotopic distance between two vibrations influences the extent to which the perception of frequency is sensitive to rms velocity. The 20 subjects (7 females) were aged between 18 and 33, and two were left handed. They compared the frequency of two vibrations separated by 500 msec, this being within the interval range at which differences between same-finger and opposite-finger comparisons are evident [6], [13]. The vibrations had either matched amplitude or matched rms velocity. The experiment additionally manipulated the location of the two vibrations: they were either delivered to the same or opposite index fingers. The subjects were tested with 4 blocks (each block comprised of two interleaved staircases, one with matched-amplitude vibrations and one with matched-energy vibrations). In two blocks, both vibrations were presented to the same location (either both on the left or both on the right index finger, randomly intermixed); in the other two blocks, one vibration was presented to the left index finger and the other vibration to the right index finger (randomly ordered). The blocks were ordered in an ABAB sequence, with the first block counterbalanced between subjects.

Experiment 4 examined the effect of noise on judgments of vibration frequency, and compared this with the impact of matching rms velocity, using a 2×2 factorial design. The 16 participants (8 females) were aged 18 to 26, and two were left handed. As in Experiments 2 and 3, they compared the frequency of vibrations that had either the same amplitude or the same rms velocity. Additionally, noise was added to the temporal structure of the two vibrations on half the trials. Noisy vibrations were constructed by adding independent Gaussian-distributed values, of positive or negative sign, to each cycle of the sine wave (see Figure 4). We added 20% noise, meaning that the standard deviation (SD) of the cycle lengths within the vibration equaled ⅕ of the base cycle length. For example, a 40 Hz vibration was comprised of cycles with mean length of 25 msec and SD of 5 msc. The subjects were tested in 4 blocks, each block containing two interleaved staircases. In one block, all vibrations were periodic (ie, without noise), and one staircase contained matched-amplitude vibrations while the other contained matched-velocity vibrations (exactly as in Experiment 3). A second block also contained one staircase with matched-amplitude vibrations and a second staircase with matched-velocity vibrations, but in this case all vibrations were noisy. The other two blocks reversed these assignments: One block contained all matched-amplitude vibrations, with one staircase of periodic vibrations and one staircase of noisy vibrations; the other block also consisted of a staircase with periodic vibrations and a staircase with noisy vibrations, but all vibrations had matched energy. The order of these four blocks was counterbalanced between subjects according to a latin square. Thus the subjects were tested twice with periodic matched-amplitude vibrations, twice with noisy matched-amplitude vibrations, twice with periodic matched-velocity vibrations, and twice with noisy matched-velocity vibrations.

Experiments 5 and 6 investigated whether the effect of noise on frequency discrimination is influenced by the somatotopic distance between the two vibrations. In Experiment 5, there were 10 subjects (6 females) aged 18 to 21 and one was left handed; in Experiment 6 there were 10 subjects in (3 females) aged 18 to 37 and three were left handed. They compared vibrations, separated by 500 msec, with either equal amplitude (and thus differing rms velocity; Experiment 5) or matched rms velocity (Experiment 6). Two interleaved staircases were run in a single block; the trials alternated between the two staircases. One staircase measured the threshold for comparing vibrations containing 20% temporal noise, as in Experiment 4, the other staircase measured the threshold for periodic (noiseless) vibrations. Each subject was tested with two such blocks (in counterbalanced order): in one block (“same-finger” condition) the first vibration on each trial was presented to the right or left index finger (by random allocation) and the second vibration was presented to that same finger; in the other block (“opposite-finger” condition) the first vibration on each trial was presented on the right or left index finger and the second vibration was presented on the other finger (left or right). A full testing session took 40 to 60 min.
